# The effect of tadalafil therapy on kidney damage caused by sepsis in a polymicrobial septic model induced in rats: a biochemical and histopathological study

**DOI:** 10.1590/S1677-5538.IBJU.2016.0075

**Published:** 2017

**Authors:** Erdal Benli, Sema Nur Ayyildiz, Selma Cirrik, Sibel Koktürk, Abdullah Cirakoglu, Tevfik Noyan, Ali Ayyildiz, Cankon Germiyanoglu

**Affiliations:** 1 Department of Urology, Faculty of Medicine, Ordu University, Ordu, Turkey;; 2 Department of Biochemistry, Faculty of Medicine, Ordu University, Ordu, Turkey;; 3 Department of Physiology, Faculty of Medicine, Ordu University, Ordu, Turkey;; 4 Department of Histolology, Faculty of Medicine, Ordu University, Ordu, Turkey;; 5 Department of Urology, Faculty of Medicine, Ondokuz Mayis University, Samsun, Turkey

**Keywords:** Acute Kidney Injury, Tadalafil, Kidney Failure, Chronic, Renal Insufficiency

## Abstract

**Introduction:**

Sepsis is an inflammatory reaction to bacteria involving the whole body and is a significant cause of mortality and economic costs. The purpose of this research was to determine whether tadalafil exhibits a preventive effect on sepsis in a septic model induced in rats with cecal ligation and puncture (CLP).

**Materials and Methods:**

Rats were randomly separated into groups, 10 rats in each: (i) a sham (control) group, (ii) an untreated sepsis group, (iii) a sepsis group treated with 5mg/kg tadalafil and (iv) a sepsis group treated with 10mg/kg tadalafil. A polymicrobial sepsis model was induced in rats using CLP. Rats were sacrificed after 16h, and blood and kidney tissues were collected for biochemical and histopathological study.

**Results:**

Levels of the inflammatory parameter IL-6 decreased significantly in the sepsis groups receiving tadalafil in comparison with the untreated sepsis group (p<0.05). In terms of histopathology, inflammation scores investigated in kidney tissues decreased significantly in the sepsis groups receiving tadalafil compared to the untreated sepsis group (p<0.05). In addition, levels of creatinine and cystatin C measured in septic rats receiving tadalafil were lower by a clear degree than in septic rats (p<0.05).

**Conclusion:**

In this study, tadalafil exhibited a preventive effect for sepsis-related damage by suppressing inflammation in serum and kidney tissue of septic rats in a polymicrobial sepsis model induced with CLP.

## INTRODUCTION

Sepsis, defined as a systemic inflammatory reaction to bacterial agents, continues to be a significant cause of mortality and economic burdens (1). Although the cause is not fully understood, according to the most commonly accepted view, an immune response is thought to be initiated in association with various endotoxins, pro-inflammatory cytokines and mediators resulting from monocyte activity against bacteria (2). Injury that may occur due to sepsis is associated with the severity of the response. When the inflammatory response initiated is prolonged or extended, several pro-inflammatory cytokines and mediators, and particularly free oxygen radicals (ROS), may become involved and cause organ losses and death (3). Impairment of the oxidant-antioxidant balance resulting from an increase in oxidants is responsible for the development of these injuries. MPO and MDA are often used as oxidant parameters and SOD and CAT activity as antioxidant parameters in studies. Ritter et al. reported that CAT activity is related to sepsis mortality (4).

Despite advances in technology and medicine, the prevalence of sepsis and the mortality it causes are increasing. Sepsis is responsible for approximately 30% of hospital deaths in America, and is the 11^th^ most common cause of death (5). Extensive research is therefore being performed across the World aimed at the treatment of sepsis. Several drugs have been tested for therapeutic purposes in sepsis models.

Tadalafil which has a long-lasting effect inhibits phosphodiesterase enzyme-5 (PDE5) which hydrolyzes cGMP. Therefore, inhibition of PDE5 with tadalafil increases the level of both cGMP and nitric oxide in medium (6). PDE5 enzyme inhibition has been shown to increase local levels of cAMP and cGMP, which cause expansion in the vascular system (7). Additionally, PDE5 inhibitors cause the activation of the enzymes endothelial nitric oxide synthase (eNOS) and inducible nitric oxide synthase (iNOS), which are involved in the synthesis of nitric oxide (NO). cAMP and cGMP that have important roles in several intracellular events such as inflammation are significant second messengers (8). Various studies concerning PDE5 inhibitors have reported different effects. These include intracellular NO production inhibiting platelet aggregation (9), improved renal blood flow in a model of partial ureteral obstruction induced in rats (10) and protection against tubular apoptosis (11).

Tadalafil increases levels of cGMP/NO, which is involved in several physiological processes. It can reduce inflammation in the vascular system and kidney tissue by reducing inflammatory response occurring during the course of sepsis. Our hypothesis was that tadalafil would protect against sepsis by suppressing inhibition in the vascular system and kidney tissue in a polymicrobial sepsis model.

This study investigated whether tadalafil would protect against sepsis-related injury in the kidney and vascular system in a polymicrobial sepsis model.

## MATERIALS AND METHODS

### Animals

Forty male albino Wistar rats with initial weights of 225-250gr were used in this study. Following approval from Ondokuz Mayıs University Faculty of Medicine local ethical committee, the animals were taken from the Samsun Experimental Animals Research and Application Center (EARAC). Rats were housed in steel cages, with a maximum 5 animals to a cage, throughout the experiment. They were allowed ad libitum access to food and water. The room in which they were housed was adjusted to a 12h light, 12h dark cycle. In addition, a split air conditioner was used to endure that the room temperature did not exceed 22±1ºC. (Temperature was measured with a thermometer and humidity with a hygrometer). Ventilation was provided by a room aspirator.

### Experimental plan

Rats were randomly separated into groups, 10 rats in each. Groups were established as follows; Group 1 sham (control; n: 10), Group 2: untreated sepsis (cecal ligation and puncture (CLP)) group, Group 3: sepsis group receiving 5mg/kg tadalafil (CLP+TAD 5mg; n: 10) and Group 4: sepsis group receiving 10mg/kg tadalafil (CLP+TAD 10mg; n: 10). Each group was placed into separate cages following this procedure.

### Sepsis model

The CLP polymicrobial sepsis model was established by ligating the distal part of the rat cecum and two-hole was punctured in the cecum. Ketamine hydrochloride (i.m. 60mg/kg) and xylazine (i.m. 10mg/kg) were used for pre-surgical anesthesia. Once the surgical area had been cleaned, an abdominal incision was made. The cecum was isolated and ligated distally to the ileocecal valve with 3/0 silk. The part of the cecum located distally to the ligation was pierced in two places using a 22 gauge needle and then replaced in the cavity. Then, the surgical site was closed properly with an absorbable suture.

Once the wound had been closed, 1% lidocaine was injected into the incision line for analgesia. In the sham group, laparotomy only was performed, and the cecum was merely raised and then replaced, with no piercing. The procedure was concluded with anatomical closure. All rats were given subcutaneous 2mL/100g saline solution for fluid solution during surgery and for 6h post-surgically. Once the procedure was completed, tadalafil suspended in saline solution was administered by gavage in doses of 5 or 10mg/kg. Taking account of variations in blood flow occurring during sepsis and the related variations in stomach and intestinal absorption, high tadalafil doses of 5 and 10mg/kg were used. These doses have been previously used in the literature and are known to be used reliably in the literature. The same amount of saline was given to the sham and CLP groups, which did not receive tadalafil. Access to food, but not water, was prevented for 16 after surgery. On the 16^th^ hour postoperatively, rats were sacrificed by exsanguination under anesthesia and blood and tissue specimens were collected. Studies have shown that depending on the size of the incision opened in the intestines to induce sepsis, frequently rats die of sepsis within 12-20 hours of incision. In studies, it is known that hyperdynamic, hyperinsulinemic, high blood lactate levels and hypermetabolic situations occur about 10 hours after CLP. Without treatment in this period, the majority die. Studies by Hubbard et al. showed that nearly 16 hours after CLP rats were hypoglycemic, hypoinsulinemic, had hypodynamic blood circulation and increased blood lactate levels. As a result, it was necessary to provide treatment within this period and to administer a higher dose than the classic dose. Thus the mortality rates may be reduced. The kidneys were quickly extracted and washed in iced saline. Half of each kidney was then stored at-80°C for biochemical investigation, while the other half was placed in 10% formalin solution for histopathological study (12-15).

### Biochemical analyses in tissue and serum

After being kept at room temperature for 30 min, blood specimens placed into gel-containing tubes were centrifuged at 3000g for 15 min. Serum samples separated from the centrifuged blood were stored as aliquots with left kidney tissue at-80^o^ C until analysis.

### Creatinine assay

Creatinine measurements in serum were performed using Abbott brand commercial creatinine (Creatinine Lot No: 78067UN14) kits on an Abbott Architect C8000 autoanalyzer in the Ordu University Faculty of Medicine biochemistry laboratory.

### Cystatin-C measurement

Cystatin-C measurements in serum specimens were performed using a rat-specific ELISA kit (Boster, EK1109, Lot No: 6551072708). Specimens were investigated with 1:50 dilution. Results were read at a 450nm wavelength on an ELISA reader (Biotek, ELX 800).

### Malondialdehyde assay

Tissue MDA measurement was performed using rat-specific ELISA kits (Bluegen: E02M0023, Lot No: 20140825). Prior to homogenization, tissues were washed in PBS buffer (0.02mol/L, pH 7.0-7.2) to remove all blood. Homogenization was then performed (100mg tissue/1mL PBS). The tissue homogenates were frozen and thawed twice and then centrifuges at 1500g for 15 min. The kit prospectus was applied with 1:10 dilution in the supernatants. The results were read at a 450nm wavelength. These were standardized with the mg protein level in the homogenate. MDA levels in serum specimens were measured using the same kit without dilution.

### Superoxide dismutase activity measurement

SOD activity in rat kidney tissue and serum was measured using spectrophotometry (CAYMAN; 706002). Blood was removed by washing the tissue with PBS, and specimens were then placed in homogenization buffer (20mM HEPES, 1mM EGTA, 210mM mannitol, 70mM sucrose; ph 7.2). The homogenates obtained were centrifuged at 1550g for 5 min (4^o^ C). These were used for SOD measurement following 1:250 dilution. Results were read at a 450nm wavelength. Rat serum specimens were studied using the same method with 1:50 dilution.

### Myeloperoxidase activity measurement

MPO measurement in rat kidney tissue and serum was performed using competitive enzyme immunoassay ELISA kits (Bluegene; E02M0032, Lot No: 20140825). Prior to homogenization, tissues were washed in PBS buffer (0.02mol/L, pH 7.0-7.2) to remove all blood. Homogenization was then performed (100mg tissue/1mL PBS). The tissue homogenates were frozen and thawed twice and then centrifuged at 1500g for 15 min. The supernatant was diluted 1:100 and the results read at 450nm on an ELISA reader. Serum specimens were studied with 1:10 dilution.

### Catalase activity measurement

CAT activity was measured using spectrophotometry with a commercial kit (Cayman; 707002). Tissues placed in homogenization tissue (50mM potassium phosphate, 1mM EDTA, pH 7.0) were homogenized in ice (5mL buffer/1gram tissue) and then centrifuged at 10.000g for 15 min. Enzyme activity was measured by diluting the supernatants obtained 250 times. Results were read at a 540nm wavelength. Rat sera were studied without dilution.

### Interleukin-6 measurement in serum

IL-6 assay in rat serum was performed using a rat-specific IL-6 ELISA kit (Boster; EK0412, Lot No: 1331047708). Results were read at a 450nm wavelength.

### Serum procalcitonin measurement

Serum PCT measurements were performed on a VidasBioMerieux device using Vidas Brahms PCT (Lot: 1003569530) kits.

### Tissue protein measurement

Protein assay in homogenates prepared as described above was performed spectrophotometrically using the Bradford method (Thermoscientific; 23200). Measurements in specimens diluted 1:20 were performed at 595nm, and the protein level in a specimen was determined based on a standard.

### Histopathologic evaluation of kidney tissue

The kidney tissues were fixed with buffered 4% paraformaldehyde and embedded in paraffin. The paraffin-embedded kidney specimens were cut into 5µm sections and processed for anti-Macrophage antibody (MAC387, sc-66204, Santa Cruz Biotechnology, Inc.) immunohistochemistry and Masson trichrome staining.

### Masson’s Trichrome Stain Procedure

The slices were processed for assessment of the structural alterations in the kidney tissue (tubule damage, vacuolization, tubular dilation and cast structure, infiltration, interstitial changes and renal corpuscle morphology) with Masson’s trichrome staining. The deparaffinized sections were treated with Weigert’s iron hematoxylin, hydrochloric acid, ponceau acid fuchsin, phosphomolybdic acid, phosphotungstic acid and aniline blue solutions. The sections were washed in distilled water and rinsed in glacial acetic acid solution to take off excessive the aniline blue. Finally, the sections were processed with ethanol and xylene, followed with mounting media (16).

### Mac387 Antibody Staining

The sections were stained with Staining System (sc-2017, Santa Cruz Biotechnology, Inc.) for the Mac387 (1:200 dilution) antibody. The paraffin sections were deparaffinized in xylene. The sections were treated with hydrogen peroxide solution for remove the endogen peroxidase activity. The sections were dehydrated with alcohol series, processed with the normal serum. The sections were treated by the primer antibody on overnight at+4°C. The sections were treated by the secondary antibody and then with enzyme reagent. The sections were visible using AminoethylCarbazole (AEC) as chromogen and mounted for analysis. The negative controls of sections processed without the primary antibody (16). Eventually, the stained sections were photographed with a camera (Leica DFC295) put on a microscope (Leica DM2500) and saved as Tagged Image File Format (TIFF).

### Inflammation-based score for kidneys

The sections were acquired systematically and sampled randomly. They were scored depending on the quantity of inflammation in the kidney as follows: 0: no cells; 1: a few cells; 2: many cells and 3: numerous cells in the kidney.

**Figure 1:**
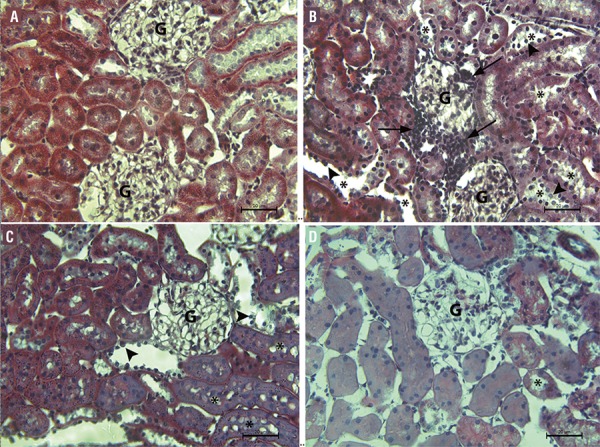
(A-D) - Representative photomicrographs of Masson’s trichrome staining of the renal cortex in the sham group (a) and sepsis group (b) 5mg/kg (c) and 10mg/kg tadalafil+sepsis groups (d). Arrows indicate areas of inflammatory cell infiltration.

**Figure 2:**
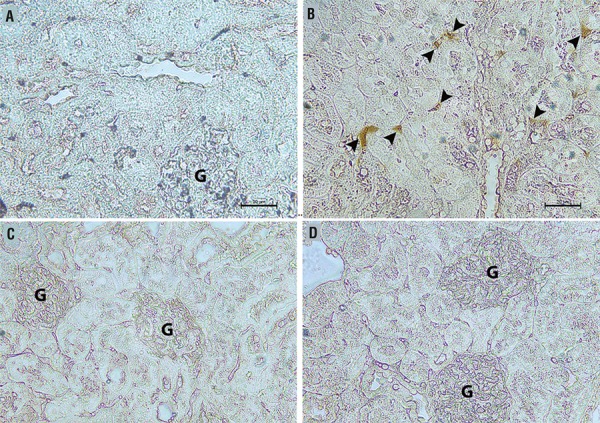
(A-D) - Representative photo micrographs of Mac387 immuno histochemistry of the renal cortex in the sham group (a) and sepsis group (b) 5mg/kg (c) and 10mg/kg tadalafil+sepsis groups (d).(a) and sepsis group (b) 5mg/kg (c) and 10mg/kg tadalafil+sepsis groups (d). Arrows indicate areas of inflammatory cell infiltration.

The sham group kidneys exhibited normal-appearing glomeruli, tubules and interstitium when stained with Masson’s trichrome. The kidney injury in the untreated sepsis group was significantly greater compared with the sham group and the 5 and 10mg/kg tadalafil+sepsis groups (P<0.05). In the untreated sepsis group, glomerulus deformities, tubular injury and inflammatory cell infiltration in the glomeruli and interstitium were observed. Tubular injury, defined as tubular dilatation, vacuolization, lumen enlargement, epithelial flattening and cell sloughing, was also seen in the untreated sepsis group. Tubular dilatation, vacuolization and epithelial flattening decreased and no inflammatory cell infiltration was observed in the glomeruli or interstitium in the 5 and 10mg/kg tadalafil+sepsis groups (Figure-1). Mac387 immunoreactivity scores in the untreated sepsis group were significantly higher compared with those in the sham, 5 and 10mg/kg tadalafil+sepsis groups (P<0.05). Mac387 immunoreactivity scores in the 5 and 10mg/kg tadalafil + sepsis groups were not significantly different from those in the sham group. There was also no significant difference between immunoreactivity scores in the 5 and 10mg/kg tadalafil + sepsis groups (Figure-2).

Arrow heads indicate flattened tubular epithelial cells in dilated tubules. * indicate tubular dilatation and vacuolization. **G**, glomerule

Arrow heads indicate Mac387 positivecells. **G** = glomerule

### Statistical analysis

The data were firstly analyzed using Levene’s test and the Shapiro-Wilk test for equality of variance and for normality assumption, respectively. Normally distributed continuous data (CAT, SOD, MPO, MDA, cystatin C and creatinine) were expressed as sample size, mean with standard deviation and secondly analyzed by using one-way ANOVA and the Tukey test. Finally, when homoscedasticity and normality were not confirmed by the tests (P<0.05) for IL-6, the Kruskal-Wallis H test and Steel-Dwass multiple comparisons were used to determine the differences among the four groups. Non-normally distributed data were expressed as sample size, mean, standard deviation, median values, interquartile range (IQR), minimum and maximum values. If the p-value is under 0.05, results are considered statistically significant.

## RESULTS

Oxidant and antioxidant activity were measured in serum and kidney tissue in order to determine the level of injury occurring during the septic process. MPO and MDA activities were measured as oxidant markers and SOD and CAT activities as antioxidant markers. SOD, CAT, MPO and MDA measurement results from kidney tissue are shown in Table-1 and results from serum are given in Table-2.

**Table 1:** Effect of tadalafil on CAT, SOD, MPO and MDA levels identified in renal tissue with sepsis.

	Sham group	Sepsis group	Sepsis+TAD (5mg)	Sepsis+TAD (10mg)
n	10	10	10	10
**CAT** (nmol/dk/ugprot)	1.93±0.61	1.64±0.37	2.22±1^a*^	1.98±0.48^a*^
**SOD** (U/mg protein)	2.69±2.29	1.64±0.24	1.98±0.7^a*^	2.67±2.7^a*^
**MPO** (ug/mg protein)	54.48±35.64	57.08±40.54	38.89±24.55^a*^	34.91±21.08^a*^
**MDA** (ng/mg protein)	0.42±0.07	0.45±0.08	0.43±0.13^a*^	0.44±0.07^a*^

Values are mean±standard deviation in each group.

**n** = number of rats; ^**a**^ = compared with sepsis group; *p>0.05.

**Table 2:** Effect of tadalafil on CAT, SOD, MPO and MDA levels in rat serum with sepsis.

	Sham group	Sepsis group	Sepsis+TAD (5mg)	Sepsis+TAD (10mg)
**T**	10	10	10	10
**CAT** (umol/dk/mL)	0.54±0.28	0.51±0.28	0.90±0.65^a*^	0.73±0.38^a*^
**SOD** (U/mL)	2.78±2.78	1.53±0.26	1.65±0.35^a*^	1.57±0.42^a*^
**MPO** (ng/mL)	16.5±9.05	16.8±13.5	14.43±7.31^a*^	14.02±9.84^a*^
**MDA** (ng/mL)	3.42±0.99	3.54±0.79	3.16±0.58^a*^	3.29±0.74^a*^

Values are mean±standard deviation in each group.

^**a**^ = compared with sepsis group; *p>0.05.

IL-6 levels used as a marker of inflammation were measured in serum. These were significantly lower in the groups receiving 5 and 10mg/kg tadalafil than in the untreated sepsis group (p<0.05) (Table-3). Creatinine and cystatin C levels were measured in serum in order to evaluate kidney functions. Creatinine and cystatin C levels measured in serum were significantly higher in the untreated sepsis group compared to the sham group (P<0.001) (Table-4). In addition, cystatin C and creatinine levels were significantly lower in the groups receiving 5 and 10mg/kg tadalafil than in the untreated sepsis group (p<0.001). There was no difference in effect between 5 and 10mg/kg doses.

**Table 3:** Variations in IL-6 levels between the groups.

Groups	n	Mean	SD	Median	IQR	Min	Max	χ^2^value	P-value
Sham group	10	4.46	1.95	4.03^c^	2.64	1.86	8.68	33.71	<0.001
Sepsis group	10	27826.67	33993.74	2469.14^a^	66886.76	1053.5	70207.5		
Sepsis+TAD (5mg)	10	53.5	32.79	40.9^b^	43.74	17.98	114.5		
Sepsis+TAD (10mg)	10	40.2	32.25	31.45^b^	29.42	114.5	107.8		

**a,b,c** = indicate differences between the groups (p<0.01).

**Table 4:** Effect of tadalafil on cystatin C and creatinine levels in rats serum with sepsis.

	Sham group	Sepsis group	Sepsis+TAD (5mg)	Sepsis+TAD (10mg)
**n**	10	10	10	10
Cystatin C (ng/mL)	511.17±57.72	988.27±288.44	527.58±92.851^a*^	575.31±41.931^a*^
Creatinine (mg/dL)	0.48±0.06	0.56±0.14	0.51±0.03^a***^	0.50±0.02^a**^

Values are mean±standard deviation in each group.

^**a**^ = compared with sepsis group; *p<0.001, **p<0.01,***p<0.05.

### Histopathological results

The sham group kidneys exhibited normal-appearing glomeruli, tubules and interstitium when stained with Masson’s trichrome. The kidney injury in the untreated sepsis group was significantly greater compared with the sham group and the 5 and 10mg/kg tadalafil+sepsis groups (P<0.05). In the untreated sepsis group, glomerulus deformities, tubular injury and inflammatory cell infiltration in the glomeruli and interstitium were observed. Tubular injury, defined as tubular dilatation, vacuolization, lumen enlargement, epithelial flattening and cell sloughing, was also seen in the untreated sepsis group (Table-5). Tubular dilatation, vacuolization and epithelial flattening decreased and no inflammatory cell infiltration was observed in the glomeruli or interstitium in the 5 and 10mg/kg tadalafil+sepsis groups (Figure-1). Mac387 immunoreactivity scores in the untreated sepsis group were significantly higher compared with those in the sham, 5 and 10mg/kg tadalafil+sepsis groups (P<0.05). Mac387 immunoreactivity scores in the 5 and 10mg/kg tadalafil+sepsis groups were not significantly different from those in the sham group. There was also no significant difference between immunoreactivity scores in the 5 and 10mg/kg tadalafil+sepsis groups (Figure-2).

**Table 5:** Histopathological variations in renal tissue.

	Sham group	Sepsis group	Sepsis+TAD (5mg)	Sepsis+TAD (10mg)
n	6	6	6	6
Mean inflammation score	0	3	1^a*^	1^a*^
Tubular degeneration	0	3	2^a*^	2^a*^

**n** = number of rats; ^**a**^ = Compared with CLP group; *p<0.05

## DISCUSSION

Both biochemical and histopathological analysis in serum and kidney tissue revealed that tadalafil exhibits a protective effect against kidney injury in sepsis. This protective effect may derive from suppression of oxidative stress and inflammation involved in tissue injury caused by polymicrobial sepsis. Its anti-inflammatory effect was also proved by a decrease in inflammatory scores in kidney tissue at histopathological analysis. Tadalafil may exhibit this effect by increasing the levels of cGMP and NO that decrease in association with cytokines and mediators occurring during the septic process.

There is a known association between severity and duration of inflammatory response against a bacterial agent and the resulting tissue injury (17). The bacterium encounters macrophages where it enters the body, and these seek to eliminate it. If the bacterium cannot be quickly eliminated, the inflammatory process is converted into a systemic response through pro-inflammatory mediators and cytokines such as tumor necrosis factor (TNF-α), interleukin-1 (IL-1) and IL-6 released by the macrophages (18). Neutralization of mediators released from macrophages in peritonitis induced in a rat model has been shown to prolong survival and reduce organ loss (19). In our study, levels of the pro-inflammatory mediator IL-6 decreased in the groups receiving tadalafil compared to the untreated sepsis group. In addition, inflammatory scores in kidney tissue decreased at pathological analysis. This shows that tadalafil suppresses the inflammatory process. Kermarrec et al. reported that nitric oxide suppressed inflammation in a model of endotoxic lung injury induced in rats, and this was compatible with our own data (20). Tadalafil may exhibit this anti-inflammatory effect by increasing cGMP/NO levels.

The production of oxygen radicals with pro-inflammatory effects such as endothelial injury, accumulation of oxidants and the formation of chemotactic factors increase during sepsis (21). Other significant effects include the inactivation of the enzyme guanylyl cyclase (sGC), which plays an important role in the synthesis of NO and thus a decrease in cGMP/NO levels (22). If sepsis cannot be halted, the inflammatory process becomes increasingly severe and results in organ dysfunction (23). The kidney is the principle vital organ injured. Sepsis is the underlying cause in approximately 50% of cases developing acute kidney failure, and approximately 70% of these cases are fatal. Protection of the kidneys in sepsis is reported to be of vital importance in terms of survival (24).

The protective effect of tadalafil in septic rats in this study, and particularly the protective effect in terms of kidney functions, was shown by creatinine and cystatin C levels measured in serum and at histopathological analysis in renal tissue. Özbek et al. reported, in agreement with our results, that tadalafil exhibited a protective effect against kidney damage (25). This may be attributed to the cGMP/NO level that decreases during sepsis being protected by tadalafil. One previous study showed that NO levels in kidney tissue decrease in renal injury (26). In the light of all these findings, cGMP/NO has a clear suppressing effect on the inflammatory process. The decrease in IL-6 levels measured in the groups receiving tadalafil compared to the untreated sepsis group also corroborates this. Muzaffar (27) and Vignozzi et al. (28) also reported the anti-inflammatory effects of PDE5 inhibitors.

Another system that is compromised during the inflammatory process is the coagulation system. This has been shown in studies involving septic animals and humans (29). One of the most commonly seen coagulation disorders, disseminated intravascular coagulation (DIC), is particularly observed in cases of severe sepsis and has been shown to be an independent risk factor for mortality in these patients (30). Yoshimoto et al. reported that intracellular NO production inhibited aggregation and caused antithrombotic effects (31). This may constitute another of the beneficial effects of tadalafil in sepsis. In association with this, we think that one of the beneficial effects of tadalafil in sepsis is that it improves coagulation disorders impaired in sepsis. The fact that no evaluation of coagulation was performed in this study represents one of its limitations.

Very few studies have investigated the use of PDE5 inhibitors in sepsis. In one such study, Cadirci et al. investigated the effect of sildenafil in a sepsis model induced with CLP through biochemical analysis in serum and through histopathological analysis in lung and kidney tissue. At the end of the study they reported that sildenafil reduced inflammation in both kidney tissue and serum and had a protective effect against sepsis (14). Our findings in terms of renal outcomes were similar. In contrast to that study, however, we determined no difference between the untreated sepsis group and the sepsis groups receiving tadalafil in terms of antioxidant and oxidant parameters measured in serum and kidney tissue.

There are various limitations to this study. One is that coagulation parameters were not investigated. Another is that cGMP and NO levels in serum and kidney tissue were not established.

## CONCLUSIONS

In conclusion, this study shows that tadalafil has a protective effect in the kidney and vascular system in sepsis through improvement in antioxidant and oxidant parameters investigated in both serum and kidney tissue. A protective effect on kidney functions was determined both through the measurement of creatinine and cystatin C levels in serum and through pathological investigation in kidney tissue. For the protective effect against sepsis, 5mg/kg dose of tadalafil was found to be sufficient. There was no difference in effect between 5 and 10mg/kg doses. We think that these findings are important for various clinical conditions, such as sepsis, and now need to be supported by further studies. If the beneficial effects identified in animals can also be shown in humans, then tadalafil may represent a new dawn in the treatment of sepsis and erection therapy.

## References

[1] Angus DC, Linde-Zwirble WT, Lidicker J, Clermont G, Carcillo J, Pinsky MR (2001). Epidemiology of severe sepsis in the United States: analysis of incidence, outcome, and associated costs of care. Crit Care Med.

[2] Sato R, Nasu M (2015). A review of sepsis-induced cardiomyopathy. J Intensive Care.

[3] Remick DG (2007). Pathophysiology of sepsis. Am J Pathol.

[4] Ritter C, Andrades M, Frota ML, Bonatto F, Pinho RA, Polydoro M (2003). Oxidative parameters and mortality in sepsis induced by cecal ligation and perforation. Intensive Care Med.

[5] Gaieski DF, Edwards JM, Kallan MJ, Carr BG (2013). Benchmarking the incidence and mortality of severe sepsis in the United States. Crit Care Med.

[6] Cairoli C, Reyes LA, Henneges C, Sorsaburu S (2014). PDE5 inhibitor treatment persistence and adherence in Brazilian men: post-hoc analyses from a 6-month, prospective, observational study. Int Braz J Urol.

[7] Humbert M, Sitbon O, Simonneau G (2004). Treatment of pulmonary arterial hypertension. N Engl J Med.

[8] Yildirim A, Ersoy Y, Ercan F, Atukeren P, Gumustas K, Uslu U (2010). Phosphodiesterase-5 inhibition by sildenafil citrate in a rat model of bleomycin-induced lung fibrosis. Pulm Pharmacol Ther.

[9] Major TC, Handa H, Annich GM, Bartlett RH (2014). Development and hemocompatibility testing of nitric oxide releasing polymers using a rabbit model of thrombogenicity. J Biomater Appl.

[10] Ayyildiz A, Kaya M, Karaguzel E, Bumin A, Akgul T, Aklan Z (2009). Effect of tadalafil on renal resistivity and pulsatility index in partial ureteral obstruction. Urol Int.

[11] Akgül T, Huri E, Yagmurdur H, Ayyildiz A, Ustün H, Germiyanoğlu C (2011). Phosphodiesterase 5 inhibitors attenuate renal tubular apoptosis after partial unilateral ureteral obstruction: an experimental study. Kaohsiung J Med Sci.

[12] Hubbard WJ, Choudhry M, Schwacha MG, Kerby JD, Rue LW, Bland KI (2005). Cecal ligation and puncture. Shock.

[13] Brewer MB, Stump AL, Holton LH, Janes LE, Silverman RP, Singh DP (2012). Effects of systemic tadalafil on skin flap survival in rats. Eplasty.

[14] Cadirci E, Halici Z, Odabasoglu F, Albayrak A, Karakus E, Unal D (2011). Sildenafil treatment attenuates lung and kidney injury due to overproduction of oxidant activity in a rat model of sepsis: a biochemical and histopathological study. Clin Exp Immunol.

[15] Coskun AK, Yigiter M, Oral A, Odabasoglu F, Halici Z, Mentes O (2011). The effects of montelukast on antioxidant enzymes and proinflammatory cytokines on the heart, liver, lungs, and kidneys in a rat model of cecal ligation and puncture-induced sepsis. ScientificWorldJournal.

[16] Topdag M, Iseri M, Topdag DO, Kokturk S, Ozturk M, Iseri P (2014). The effect of etanercept and methylprednisolone on functional recovery of the facial nerve after crush injury. Otol Neurotol.

[17] Ali A, Na M, Svensson MN, Magnusson M, Welin A, Schwarze JC (2015). IL-1 Receptor Antagonist Treatment Aggravates Staphylococcal Septic Arthritis and Sepsis in Mice. PLoS One.

[18] Boomer JS, Shuherk-Shaffer J, Hotchkiss RS, Green JM (2012). A prospective analysis of lymphocyte phenotype and function over the course of acute sepsis. Crit Care.

[19] Ness TL, Hogaboam CM, Strieter RM, Kunkel SL (2003). Immunomodulatory role of CXCR2 during experimental septic peritonitis. J Immunol.

[20] Kermarrec N, Zunic P, Beloucif S, Benessiano J, Drouet L, Payen D (1998). Impact of inhaled nitric oxide on platelet aggregation and fibrinolysis in rats with endotoxic lung injury. Role of cyclic guanosine 5’-monophosphate. Am J Respir Crit Care Med.

[21] Andrades ME, Ritter C, Dal-Pizzol F (2009). The role of free radicals in sepsis development. Front Biosci.

[22] Parrillo JE (1993). Pathogenetic mechanisms of septic shock. N Engl J Med.

[23] Kharb S, Singh V, Ghalaut PS, Sharma A, Singh GP (2000). Role of oxygen free radicals in shock. J Assoc Physicians India.

[24] Schrier RW, Wang W (2004). Acute renal failure and sepsis. N Engl J Med.

[25] Özbek K, Ceyhan K, Koç F, Söğüt E, Altunkaş F, Karayakali M (2015). The protective effect of single dose tadalafil in contrast-induced nephropathy: an experimental study. Anatol J Cardiol.

[26] Devrim E, Cetin M, Namuslu M, Ergüder IB, Cetin R, Durak I (2009). Oxidant stress due to non ionic low osmolar contrast medium in rat kidney. Indian J Med Res.

[27] Muzaffar S, Shukla N, Srivastava A, Angelini GD, Jeremy JY (2005). Sildenafil citrate and sildenafil nitrate (NCX 911) are potent inhibitors of superoxide formation and gp91phox expression in porcine pulmonary artery endothelial cells. Br J Pharmacol.

[28] Vignozzi L, Gacci M, Cellai I, Morelli A, Maneschi E, Comeglio P (2013). PDE5 inhibitors blunt inflammation in human BPH: a potential mechanism of action for PDE5 inhibitors in LUTS. Prostate.

[29] Nickel KF, Laux V, Heumann R, Degenfeld G von (2013). Thrombin has biphasic effects on the nitric oxide-cGMP pathway in endothelial cells and contributes to experimental pulmonary hypertension. PLoS One.

[30] Gaieski DF, Edwards JM, Kallan MJ, Carr BG (2013). Benchmarking the incidence and mortality of severe sepsis in the United States. Crit Care Med.

[31] Yoshimoto H, Suehiro A, Kakishita E (1999). Exogenous nitric oxide inhibits platelet activation in whole blood. J Cardiovasc Pharmacol.

